# Biomarkers for site-specific response to neoadjuvant chemotherapy in epithelial ovarian cancer: relating MRI changes to tumour cell load and necrosis

**DOI:** 10.1038/s41416-020-01217-5

**Published:** 2021-01-04

**Authors:** Jessica M. Winfield, Jennifer C. Wakefield, James D. Brenton, Khalid AbdulJabbar, Antonella Savio, Susan Freeman, Erika Pace, Kerryn Lutchman-Singh, Katherine M. Vroobel, Yinyin Yuan, Susana Banerjee, Nuria Porta, Shan E. Ahmed Raza, Nandita M. deSouza

**Affiliations:** 1 Cancer Research UK Cancer Imaging Centre, Division of Radiotherapy and Imaging, The Institute of Cancer Research, 123 Old Brompton Road, London, SW7 3RP UK; 2 MRI Unit, Royal Marsden NHS Foundation Trust, Downs Road, Sutton, Surrey SM2 5PT UK; 3 Cancer Research UK Cambridge Institute, Cambridge, CB2 0RE UK; 4 Addenbrooke’s Hospital, Cambridge University Hospitals NHS Foundation Trust, Hills Road, Cambridge, CB2 0QQ UK; 5 Department of Oncology, University of Cambridge, Cambridge, CB2 0XZ UK; 6 Centre for Evolution and Cancer, The Institute of Cancer Research, London, UK; 7 Division of Molecular Pathology, The Institute of Cancer Research, London, UK; 8 Department of Pathology, Royal Marsden NHS Foundation Trust, Fulham Road, London, SW3 6JJ UK; 9 Department of Radiology, Addenbrooke’s Hospital, Cambridge University Hospitals NHS Foundation Trust, Hills Road, Cambridge, CB2 0QQ UK; 10 Swansea Gynaecological Oncology Centre, Swansea Bay University Health Board, Singleton Hospital, Swansea, SA2 8QA UK; 11 Gynaecology Unit, Royal Marsden NHS Foundation Trust, Downs Road, Sutton, Surrey, SM2 5PT UK; 12 Clinical Trials and Statistics Unit, The Institute of Cancer Research, 123 Old Brompton Road, London, SW7 3RP UK; 13 Department of Computer Science, University of Warwick, Coventry, UK

**Keywords:** Ovarian cancer, Cancer imaging, Tumour biomarkers, Chemotherapy

## Abstract

**Background:**

Diffusion-weighted magnetic resonance imaging (DW-MRI) potentially interrogates site-specific response to neoadjuvant chemotherapy (NAC) in epithelial ovarian cancer (EOC).

**Methods:**

Participants with newly diagnosed EOC due for platinum-based chemotherapy and interval debulking surgery were recruited prospectively in a multicentre study (*n* = 47 participants). Apparent diffusion coefficient (ADC) and solid tumour volume (up to 10 lesions per participant) were obtained from DW-MRI before and after NAC (including double-baseline for repeatability assessment in *n* = 19). Anatomically matched lesions were analysed after surgical excision (65 lesions obtained from 25 participants). A trained algorithm determined tumour cell fraction, percentage tumour and percentage necrosis on histology. Whole-lesion post-NAC ADC and pre/post-NAC ADC changes were compared with histological metrics (residual tumour/necrosis) for each tumour site (ovarian, omental, peritoneal, lymph node).

**Results:**

Tumour volume reduced at all sites after NAC. ADC increased between pre- and post-NAC measurements. Post-NAC ADC correlated negatively with tumour cell fraction. Pre/post-NAC changes in ADC correlated positively with percentage necrosis. Significant correlations were driven by peritoneal lesions.

**Conclusions:**

Following NAC in EOC, the ADC (measured using DW-MRI) increases differentially at disease sites despite similar tumour shrinkage, making its utility site-specific. After NAC, ADC correlates negatively with tumour cell fraction; change in ADC correlates positively with percentage necrosis.

**Clinical trial registration:**

ClinicalTrials.gov NCT01505829.

## Background

Epithelial ovarian cancer (EOC) of tubo-ovarian origin and primary peritoneal cancer often present at an advanced stage when multiple metastatic deposits in the pelvis and abdomen are commonly seen.^1^ When primary debulking surgery is not feasible, platinum-based neoadjuvant chemotherapy (NAC) is recommended prior to interval debulking surgery (IDS) with the aim of reducing the burden of disease and enabling complete macroscopic (R0) resection, as this is strongly linked to favourable prognosis.^2,3^ However, it is now recognised that lesions may show a differential response^4^, which is related to the tissue site at which the deposits occur^5^ and the local microenvironment which may promote development of resistant metastatic clones.^6^ If it were possible to identify lesions that are likely to be poorly responsive to neoadjuvant chemotherapy, these lesions could be specifically targeted at surgical resection.

Traditionally, response in EOC has been assessed with unidimensional size measurements, which have been shown to be robust across tumour types and observer assessments.^7^ Response evaluation criteria in solid tumours (RECIST) criteria^8^ are well-established and widely used including in ovarian cancer.^9^ It is increasingly recognised, however, that early response in tumours, with induction of necrosis by cytotoxic agents, may precede changes in tumour size and requires additional imaging markers for its recognition.^10^ The apparent diffusion coefficient (ADC) derived from diffusion-weighted magnetic resonance imaging (DW-MRI) has been linked to tumour cellularity, but its relationship to the necrotic fraction within a responding tumour only has limited supporting evidence in some tumour types.^11,12^ This is largely because estimation of necrosis on pathological specimens is variable when driven by observer assessments. Also, digital analysis of the extent of necrosis^13^ has not been widely available. In this study, we aimed to measure the change in ADC metrics in EOC treated with platinum-based chemotherapy in a quality-assured/controlled multicentre trial,^14^ compare the changes in ADC between anatomic disease sites and relate them to histological measures of response (residual viable tumour and necrosis) as assessed by digital pathology.

## Methods

### Participants

Participants with newly diagnosed stage III or IV ovarian, fallopian tube or primary peritoneal cancer were recruited prospectively in a multicentre trial with multicentre research ethics committee approval (recruited 2012–2016, ClinicalTrials.gov NCT01505829, study protocol available online (Supplementary Table 1)^14^). Participants were enrolled at four hospitals (National Health Service, United Kingdom). All participants gave written informed consent. Inclusion criteria were histology/cytology-confirmed high-grade serous, endometrioid or clear cell histology, at least one solid mass >2 cm in long axis on CT or MRI and scheduled for platinum-based NAC with IDS after three or four cycles. Exclusion criteria were abdomino-pelvic radiotherapy within six months of screening, contra-indications to MRI, or receipt of an investigational compound or device within 30 days of starting treatment.

### Study design

Participants underwent baseline (pre-NAC) MRI examinations before starting chemotherapy. Two pre-NAC MRI examinations were conducted up to 7 days apart if participants were able to tolerate both examinations; only one pre-NAC examination was conducted if participants were unable to tolerate the second pre-NAC examination. Post-NAC MRI examinations were conducted after three or four cycles of chemotherapy within 8 days prior to IDS.

### MRI protocol

Slice-matched, DW-MRI, T_1_-weighted, and T_2_-weighted imaging standardised between centres (with allowances for intervendor and scanner variations^15^) covered the abdomen and pelvis in three stations (Supplementary Table 1). Regular quality assurance tests throughout the study ensured measurement stability.

### Image analysis

Images were analysed at the lead centre using in-house software (Adept, The Institute of Cancer Research, London, UK). Intermediate signal intensity masses on T_2_-weighted images with restricted diffusion identified as tumour were categorised by site (ovary, peritoneum, omentum, and enlarged lymph node by RECIST criteria). For each examination, regions-of-interest (ROIs) encompassing the whole solid lesion on all slices were drawn by region growing on computed b = 1000 smm^−2^ images^16^ by JCW (2 years’ experience with pelvic MRI) and checked by NMdS (20 years’ experience). Cystic areas were excluded by visual matching with T_2_-weighted images. Up to five target and five nontarget largest lesions per participant were analysed. Lesions were selected on pre-NAC MRI examinations and the same lesions were followed-up on post-NAC MRI examinations. ADCs were estimated by mono-exponential fitting of signal intensity at b-values 100, 500 and 900 smm^−2^. The median ADC, 25th and 75th centile (ADC_median_, ADC_25_, ADC_75_, respectively) were estimated from all fitted voxels in the ROIs for each lesion. The volume of each solid lesion was obtained by multiplying the number of voxels in the ROIs by voxel volume (range 0.013–0.016 cm^3^). For each lesion, the change in solid tumour volume and ADC after three or four cycles of NAC was expressed as percentage change from pre-NAC measurements.

### Image-guided surgical sampling

Anatomical localisation diagrams provided to participating surgeons with detailed annotations on radiologist-selected imaged target lesion location enabled matching of lesions with those identified at surgery. These matched lesions were marked with sutures at excision to identify them to the pathologist.

### Histopathology analysis

Formalin fixed tissue specimens were sectioned at three to four-millimetre intervals, embedded in paraffin and 2–3-micron sections mounted on glass slides. Haematoxylin and eosin (H&E) stained sections were reviewed by two gynaecological-oncology histopathologists in consensus (AS, KV, 15 and 3 years’ experience respectively). From each selected lesion, after review of the entire lesion, they chose a single index slide that most closely represented the residual viable tumour and necrosis across the whole lesion.

Whole H&E stained slides were digitised to a resolution of 0.26 µm per pixel (Hamamatsu NanoZoomer XR scanner, Hamamatsu, Japan). An algorithm previously trained to 92.61% accuracy on a lung model was used to identify tumour cells, differentiating them from stromal, lymphocytes and other cells such as macrophages.^17^ The proportion of viable tumour cells to total cells in the sample (tumour cell fraction) was recorded. Areas of viable tumour and necrosis outlined by AS on 20 slides were used to train a modified algorithm (MicroNet) to segment tumour and necrosis regions on the whole study sample.^18^ Algorithm training was deemed acceptable on achieving 90% validation accuracy. A pretrained H&E tissue segmentation algorithm removed background noise and artefacts.^17^ The ratios of segmented tumour or necrosis area to the whole-slide segmented tissue area were recorded as %residual tumour and %necrosis, respectively.

### Statistical analysis

Statistical analysis (NP) used commercially available software (Stata, v15.1, StataCorp, College Station, TX, USA) and GraphPad Prism for Windows, (v8.3, GraphPad Software Inc., San Diego, CA, USA). *P* values < .05 were considered statistically significant. Median, lower and upper quartiles were used to summarise imaging and histology parameters.

95% limits of agreement (LoA)^19^ were used to assess repeatability of solid tumour volume and ADC_median_ for each disease site (ovary, omentum, peritoneum and lymph nodes).

Probability density functions for voxel-wise ADC estimates were determined for pre-NAC and post-NAC measurements; the first pre-NAC examination was used in those with two examinations (commercially available software, ksdensity, Matlab, v2016a, The MathWorks Inc, Natick, MA, USA). Analysis was done on a per lesion basis rather than a cumulative voxel analysis to remove bias towards larger volumes, and the sum of probability density estimates calculated for each anatomic location.

The ADC_median_ before neoadjuvant chemotherapy (pre-NAC) between lesions that remained measurable after neoadjuvant chemotherapy (post-NAC), and those that became nonmeasurable were compared using linear mixed-effects regression models to each pre-NAC parameter, including status of lesion (measurable/nonmeasurable) as a fixed effect and per-participant random intercept effects to account for clustering within participants. For those lesions that remained measurable post-NAC, further linear mixed models were used to compare percentage change between pre- and post-NAC in solid lesion volume and ADC_median_ across disease sites (fixed effect), adjusting by baseline pre-NAC values and including per-participant random intercept. Models were fitted to logarithm-transformed data when normality assumption did not hold (checked graphically by histograms and boxplots and tested by Shapiro–Wilks test). Pairwise comparisons between disease sites are presented with adjusted differences and p-values corrected for multiplicity by Bonferroni.

The relationships between (i) post-NAC preoperative ADC_median_ and tumour cell fraction; (ii) post-NAC preoperative ADC_median_ and %residual tumour; (iii) pre/post-NAC change in ADC_median_, ADC_25_ and ADC_75_ and %necrosis were assessed using Spearman’s correlation.

## Results

### Participants and lesions

**Fig. 1:**
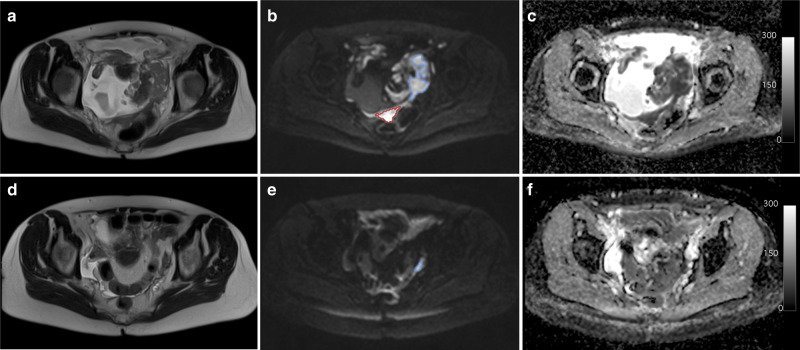
Site-specific response of EOC to neoadjuvant chemotherapy. Images in a 62-year-old woman with stage 3 high-grade serous epithelial ovarian cancer show differential response in primary and metastatic lesions: **a** axial T_2_-weighted magnetic resonance imaging (MRI) at baseline (pre-NAC), **b** corresponding axial high-b-value diffusion-weighted MRI (b = 900smm^−2^), **c** apparent diffusion coefficient (ADC) map and **d**–**f** matched sections of the same imaging series after three cycles of platinum-based chemotherapy (post-NAC preoperative). (Scalebar on the ADC map is in units of 10^−5^ mm^2^ s^−1^.) Delineation of regions of interest (ROIs) is shown in **b**, **e** for the left ovarian lesion (blue ROI) and peritoneal lesion (red ROI). The ovarian lesion remained measurable on MRI after three cycles of chemotherapy and was included in the imaging-pathology comparison, but the peritoneal lesion was nonmeasurable on MRI after three cycles of chemotherapy. MRI magnetic resonance imaging, NAC neoadjuvant chemotherapy, ADC apparent diffusion coefficient, ROI region of interest.

Fifty-two participants were enrolled. All participants were newly diagnosed and chemo naïve. Five participants were excluded, leaving 47 participants (47 women, median age 61 years, interquartile range (IQR) 57–70 years) with pre-NAC DW-MRI (Supplementary Fig. 1 and Supplementary Table 2); the five excluded participants consisted of four who were found not to have met the inclusion criteria (two had low grade final histology, one had a final diagnosis of metastatic breast cancer, one had metal hip prostheses) and one who did not undergo any MRI examinations (Supplementary Fig. 1). 47/47 participants had high-grade serous subtype. 3/47 were treated with carboplatin monotherapy, and 44/47 treated with carboplatin and paclitaxel; 5/47 also received bevacizumab (Supplementary Table 2). Two pre-NAC MRI examinations were available in 19/47 participants for repeatability assessment. 7/47 participants did not undergo post-NAC MRI examinations, leaving 40/52 participants in the final imaging analysis (Supplementary Fig. 1). Of 247 lesions at pre-NAC (50 ovarian, 114 peritoneal, 47 omental and 36 lymph node lesions), 139 lesions (40 ovarian, 50 peritoneal, 27 omental and 22 lymph nodes) remained measurable on the high b-value DW-MRI images after three or four cycles of chemotherapy in these participants (example shown in Fig. 1). Of the 40/52 participants with pre- and post-NAC DW-MRI, 7/40 participants did not have IDS, and a further 8/40 had no analysable lesions on pathology that were matched to the imaging, leaving 25/40 participants with matched lesions on imaging and pathology (Supplementary Fig. 1).

### Tumour burden and site-specific response

Site-specific repeatability for solid tumour volume was assessed in 123 lesions (20 ovarian, 52 peritoneal, 23 omental, 28 lymph node) from 19 participants. 95% LoA were −19.2 to 17.9 cm^3^ for solid elements of ovary, −5.7 to 5.4 cm^3^ for peritoneum, −43.2 to 42.3 cm^3^ for omentum and −3.2 to 2.2 cm^3^ for lymph nodes (Supplementary Table 3). Pre- and post-NAC DW-MRI was available in 40 participants (Supplementary Fig. 1). The volume of solid tumour that remained measurable in post-NAC preoperative DW-MRI is presented in Table 1 for each lesion site for pre- and post-NAC measurements. Median (lower and upper quartile) site-specific tumour burden reduction was −86.2 (−91.1, −72.6)% for solid elements of ovary, −80.1 (−87.6, −64.2)% for peritoneum, −89.4 (−97.4, −64.2)% for omentum and −80.8 (−90.6, −70.4)% for lymph nodes (Table 1). Adjusting by pre-NAC solid tumour volume, there were no statistically significant differences between lesion sites in volume reduction (linear mixed model, log-scale, global *p* = 0.14). 28 of 40 ovarian, 35 of 50 peritoneal, 19 of 27 omental and 14 of 22 lymph node lesions reduced in volume below the lower LoA.

**Table 1:** Solid lesion volume and ADC_median_ for each disease site showing changes with NAC in lesions that remained measurable after treatment. Pre-NAC values of lesions that were non-measurable after NAC are given for comparison.

	Measurable lesions presurgery (after NAC)				Nonmeasurable lesions presurgery (after NAC)	
	*n*	Pre-NAC	Post-NAC	Change/%	*n*	Pre-NAC
Solid tumour volume/cm^3^ (median [Q1–Q3])						
Ovary	40	40.0 [22.3 to 107.4]	4.8 [2.2 to 14.5]	−86.2 [−91.1 to −72.6]	10	7.2 [2.8 to 25.2]
Peritoneum	50	14.7 [4.9 to 71.7]	2.0 [1.2 to 9.9]	−80.1 [−87.6 to −64.2]	64	5.5 [2.5 to 16.0]
Omentum	27	161.2 [57.2 to 275.8]	12.7 [2.1 to 36.4]	−89.4 [−97.4 to −64.2]	20	30.0 [3.5 to 86.6]
Lymph node	22	6.1 [3.3 to 14.2]	1.0 [0.7 to 3.0]	−80.8 [−90.6 to −70.4]	14	4.1 [1.5 to 8.9]
ADC_median_/10^−5^ mm^2^ s^−1^ (median [Q1–Q3])						
Ovary	40	99 [88 to 112]	122 [108 to 139]	18.6 [7.7 to 36.2]	10	95 [86 to 102]
Peritoneum	50	101 [87 to 112]	118 [101 to 129]	11.4 [1.4 to 30.7]	64	100 [89 to 112]
Omentum	27	110 [95 to 120]	123 [106 to 131]	5.6 [−7.8 to 24.5]	20	109 [98 to 118]
Lymph node	22	102 [94 to 107]	141 [128 to 166]	38.1 [23.3 to 67.6]	14	106 [89 to 114]

The median value is shown, with lower (Q1) and upper (Q3) quartiles shown in brackets.

*n* number of lesions, *NAC* neoadjuvant chemotherapy, *ADC* apparent diffusion coefficient (where ADC_median_ is defined as the median ADC of all fitted voxels in a lesion).

### ADC metrics and site-specific response

**Fig. 2:**
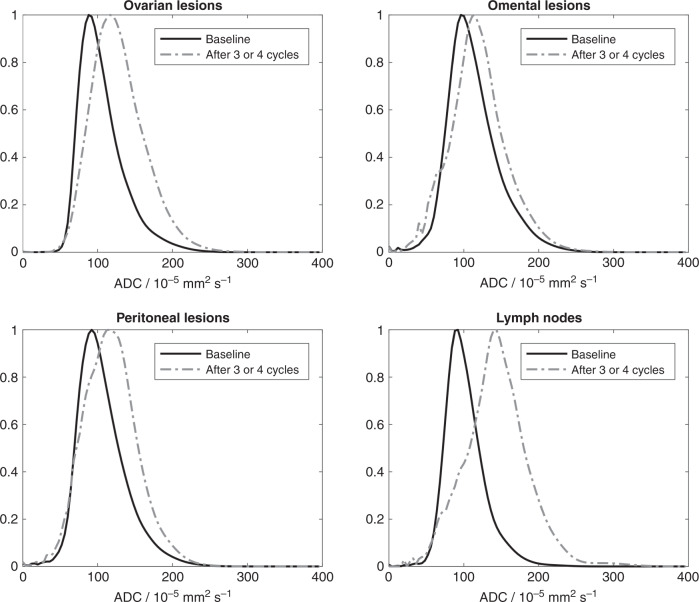
Probability density functions for ADC estimates in all lesions at each anatomic site (ovarian, omental and peritoneal lesions and lymph nodes) at baseline (pre-NAC) and after three or four cycles of treatment (post-NAC preoperative). Probability density functions have been normalised to aid comparison between pre- and post-treatment data. The same points and bandwidth were used for all lesions (bandwidths were determined for each lesion separately and the median bandwidth from all lesions estimated and applied to each lesion). ADC apparent diffusion coefficient, NAC neoadjuvant chemotherapy.

Site-specific 95% LoA for ADC_median_ were −10 to 9 × 10^−5^ mm^2^s^−1^ for solid elements of ovary, −13 to 16 × 10^−5^ mm^2^s^−1^ for peritoneum, −17 to 17 × 10^−5^ mm^2^s^−1^ for omentum and −27 to 21 × 10^−5^ mm^2^s^−1^ for lymph nodes (Supplementary Table 3). Within lesions that remained measurable post-NAC, the ADC_median_ is presented in Table 1, for each lesion site in pre- and post-NAC measurements, as well as the percentage change between pre- and post-NAC. Probability density functions for ADC estimates from all lesions at each anatomic location showed a shift towards higher ADC after NAC (Fig. 2). For the change in ADC_median_, after adjusting by pre-NAC ADC_median_ and accounting for within-participant correlation, there were differences in ADC_median_ change between peritoneal lesions and lymph node lesions (adjusted difference, diff = −23.4%, *p* = 0.001), and between omental lesions and lymph node lesions (diff = −28.7%, *p* < 0.001), but no differences between peritoneal and omental (diff = +5.2%, *p* = 0.99) or ovarian lesions (diff = −7.5%, *p* = 0.51), nor between ovarian and nodal lesions (diff = −16.0%, *p* = 0.06) or ovarian and omental (diff = 12.7%, *p* = 0.07). 28 of 40 ovarian, 24 of 50 peritoneal, 8 of 27 omental and 17 of 22 lymph node lesions increased in ADC_median_ above the upper LoA. The pre-NAC ADC_median_ of lesions that became nonmeasurable on post-NAC scans was not significantly different from those that remained measurable (Table 1), for ovarian lesions (diff = 3.9 × 10^−5^ mm^2^s^−1^, *p* = 0.58), peritoneal (diff = 4.11 × 10^−5^ mm^2^s^−1^, *p* = 0.17), omental (diff = 3.9 × 10^−5^ mm^2^s^−1^, *p* = 0.37) and lymph nodes (diff = 5.2 × 10^−5^ mm^2^s^−1^, *p* = 0.29).

### Comparison of ADC metrics with histological measures of response

**Fig. 3:**
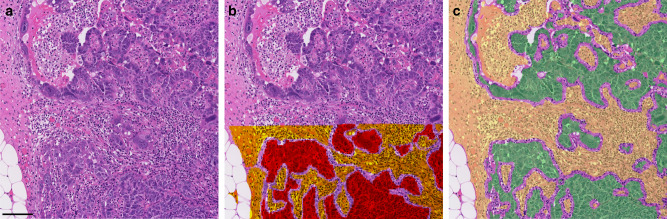
Deep learning tumour and necrosis segmentation from pathologist annotations. **a** Example of an H&E section from an omental lesion from a 63-year-old woman with stage 3 high-grade serous epithelial ovarian cancer; **b** shows tumour regions (red) and regions outlined as part of the necrosis (orange) delineated by a pathologist in the lower half of the section. The unannotated standard H&E stain is seen in the top half of the section. The same section after deep learning segmentation of the whole section (**c**), showing tumour (green) and necrosis (yellow) for comparison. The correlation between the deep learning segmentation and the ground-truth pathologist segmentation is high. The scalebar in **a** shows 100 microns. H&E haematoxylin and eosin.

In total, 99 sections (37 ovarian, 31 peritoneal, 24 omental, 7 lymph node) from 93 lesions in 25 participants were assessed on digital pathology (Fig. 3). Tumour cell fraction, %residual tumour and %necrosis median (lower quartile and upper quartile) were 50.6% (46.0% and 63.1%), 7.5% (2.2% and 19.2%) and 54.5% (35.1% and 69.6%), respectively, for ovary, 43.6% (36.0% and 50.5%), 7.8% (1.8% and 28.1%) and 56.3% (22.2% and 79.2%) for peritoneum, 41.9% (25.5% and 59.1%), 3.0% (1.2% and 17.7%) and 52.9% (36.9% and 63.6%) for omentum, and 27.9% (14.0% and 32.5%), 3.3% (1.4% and 4.7%) and 26.5% (20.4% and 74.3%) for lymph nodes.

**Fig. 4:**
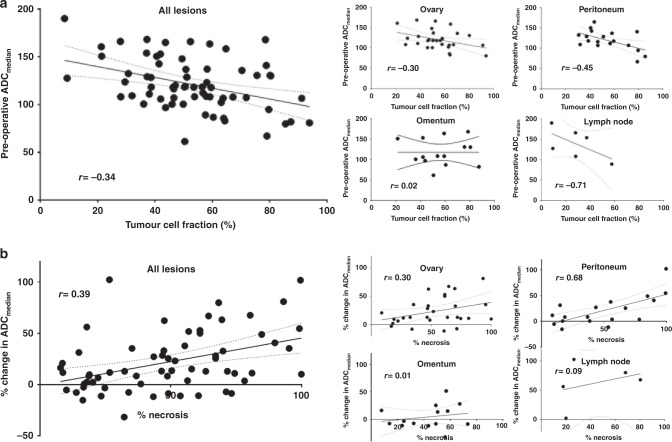
Site-specific correlations between imaging and histopathology metrics. Comparison between **a** preoperative ADC_median_ and tumour cell fraction, and **b** percentage change in ADC_median_ and %necrosis, showing all lesions considered together and ovarian, omental, peritoneal lesions and lymph nodes considered separately. r = Spearman correlation coefficient, ADC apparent diffusion coefficient (where ADC_median_ is defined as the median ADC of all fitted voxels in a lesion), tumour cell fraction = percentage of viable tumour cells to total cells in sample, %residual tumour = percentage area of whole section represented by viable tumour, %necrosis = percentage area of whole section represented by necrosis.

Of these 99 sections, matched pathology and post-NAC DW-MRI was obtained in 69 sections (29 ovarian, 20 peritoneal, 14 omental, 6 lymph node) from 65 lesions obtained from 25 participants (two large ovarian lesions were sampled in two separate areas, one large peritoneal mass was sampled in three separate areas). Table 2 shows Spearman correlation between the change in ADC metrics and histological features. When all lesions were considered together, post-NAC ADC_median_ showed negative correlation with tumour cell fraction (r = −0.34, *p* = 0.005). When considered by disease site, this held true for the peritoneum (r = −0.45, *p* = 0.05) only. The change in ADC_median_ for all sites considered together showed positive correlation with %necrosis (r = 0.39, *p* = 0.001) for the peritoneum (r = 0.68, *p* = 0.001). Illustration (Fig. 4) is restricted to statistically significant correlations.

**Table 2:** Spearman correlation of preoperative (post-NAC) ADC metrics and their change from pre-NAC values with histological measures of residual viable tumour and response.

Site	ADC_median_ vs tumour cell fraction		ADC_median_ vs %residual tumour		Percentage change ADC_median_ with %necrosis		Percentage change ADC_25_ with %necrosis^a^		Percentage change ADC_75_ with %necrosis^a^	
	r	*p*	r	*p*	r	*p*	r	*p*	r	*p*
Ovary (*n* = 29)^b^	−0.30	0.11	−0.25	0.20	0.30	0.12	0.23	0.22	0.34	0.08
Peritoneum (*n* = 20)^c^	−0.45	0.05	−0.47	0.04*	0.68	0.001*	0.71	<0.001*	0.61	0.005*
Omentum (*n* = 14)	0.02	0.93	0.24	0.42	0.01	0.97	0.25	0.41	0.00	0.99
Lymph node (*n* = 6)	−0.71	0.11	−1.00	<0.001*	0.09	0.87	0.00	0.99	0.10	0.87
All sites (*n* = 69 sections from 65 lesions)	−0.34	0.005*	−0.27	0.02*	0.39	0.001*	0.45	<0.001*	0.40	<0.001*

tumour cell fraction = percentage of viable tumour cells to total cells in sample, %residual tumour = percentage area of whole section represented by viable tumour, %necrosis = percentage area of whole section represented by necrosis.

*NAC* neoadjuvant chemotherapy, *n* number of lesions, *ADC* apparent diffusion coefficient (ADC_median_, ADC_25_ and ADC_75_ are defined as the median, 25th centile, and 75th centile of ADC estimates from all fitted voxels in a lesion, respectively),

*P  < 0.05; for lymph nodes, the sample is too small and the significant result may be due to chance.

^a^In 2 peritoneal, 1 omental and 2 lymph node lesions, ADC_25_ and ADC_75_ could not be estimated.

^b^2 lesions with >1 histology assessment.

^c^1 lesion with >1 histology assessment.

## Discussion

This study shows that in EOC/fallopian tube/primary peritoneal cancer lesions responding to NAC, there is a differential increase in ADC by anatomic site of the lesion despite a similar volume reduction of >80%. This study also confirms a site-specific correlation between the ADC changes and histological metrics (tumour cell fraction, percentage necrosis). Thus, post-NAC preoperative ADC_median_ measurement is a clinically useful method of detecting the presence or absence of viable tumour for peritoneal deposits, but not at other sites. This is useful in assessing relapsed disease, which is predominantly peritoneal. These multicentre findings also confirm pilot data where a negative correlation between epithelial cell density and diffusivity was demonstrated in 15 lesions excised at IDS in participants with primary ovarian or peritoneal cancer,^4^ and in 24 participants with prostate cancer.^20^ In orthotopic preclinical models of solid ovarian tumours, the change in ADC following docetaxel also was shown to correlate negatively with Ki67, CA125 and Bcl-2, all of which predict residual tumour burden.^21^

Although negative correlation between cell density and diffusivity is widely accepted,^22,23^ the literature is inconclusive about the effect of stroma, fibrosis and inflammatory infiltrate on ADC values indicating that they are influenced by a complex interplay of biophysical processes.^24,25^ In a tissue comprised primarily of fat, such as the omentum, the return of normal fatty stroma interspersed with residual tumour may obscure a post-treatment ADC rise. This phenomenon is well-described in adult bone marrow.^26^ This underlines the important contribution of the surrounding normal tissue to the ADC and is borne out by the omental data in this study, where ADC increases in responding lesions were lower than other sites. The differences between ADC response between peritoneal deposits and lymph nodes is of interest, as both are densely cellular tissues. It may well be that swelling and inflammatory response in lymph nodes causes a much greater ADC rise than in the fibrotic peritoneum, where inflammation and oedema is less. This requires further investigation. The study was not powered to assess the relationship of site-specific ADC change with progression-free survival (PFS) in these treatment-naïve patients, although we have previously shown that an increase in ADC of peritoneal lesions after one cycle of chemotherapy indicates improved PFS in relapsed disease.^14^

Only six lymph node lesions were analysable for imaging-pathology correlations, which limits imaging-pathology comparisons for lymph nodes alone, but we have included these for completeness in reporting this study. ADC changes in lymph nodes following chemotherapy have been reported in lymphomas,^27,28^ where abnormal nodal architecture results in variable increases in ADC; imaging-pathology studies have not been conducted as surgery is not part of the patient management. In other cancer types involving lymph nodes, treatment is usually chemoradiotherapy, where radiotherapy influences ADC rises^29,30^ due to early acute tissue injury and swelling,^31^ and cannot be easily compared to our findings.

ADC increases post-treatment have been shown to correlate with percentage necrosis in preclinical studies.^21^ A multicentre clinical trial in lung cancer concluded that presurgical ADC or change in ADC did not correlate with pathologist-assessed necrosis of resection specimens but assessment was dependent on a single pathologist reading.^32^ Clinical evidence relating ADC change following chemotherapy to subsequent necrosis may be confounded by pre-existing microscopic necrosis within the tumour; ADC change has been linked to necrosis in tumour types without much pre-existing necrosis, but not in others.^33^

Our study has several limitations. First, lesions that became unmeasurable, and therefore showed the greatest response, did not contribute to the ADC measurements. Second, lesions that could not be matched between pre-IDS MRI and histology samples were excluded, which reduced the sample size. Moreover, matching between the imaging and histology was done on the larger resectable lesions that were easily identified at surgery; smaller lesions, which may represent a bigger response, were not available for histological correlation, possibly introducing bias towards more slowly responding lesions. The selection bias was minimised by choosing up to ten lesions per participant on the baseline MRI from different anatomical sites that were representative of the participant’s disease. Third, the direction of pathological sectioning did not always exactly match the axial imaging plane. We addressed this by selecting a histological slice that most accurately represented the proportions of residual tumour and necrosis within each lesion, but this depended on detailed pathologist review, not 3D molds.^34^ To minimise error, this was done by an experienced specialist gynaecological pathologist at a national cancer centre. However, selection of a single histological slide per lesion also represented a fourth limitation. Although analysis of the entire lesion may be ideal, it was impractical to digitise and analyse large numbers of sections in each lesion. Also, the resource to do this was limited and could not be accommodated.

In conclusion, in this relatively small study in EOC, the ADC repeatability and extent of increase following treatment is anatomic site dependent. The post-NAC preoperative ADC and change in ADC is an indicator of residual viable tumour and percentage of necrosis, respectively, primarily within peritoneal deposits. When using ADC as a response indicator in EOC lesions, therefore, consideration must be given to the ADC increase compared with measurement repeatability and to the anatomic site.

## Supplementary information

Supplementary Material

## Data Availability

The data from this study are available via the Institute of Cancer Research’s XNAT imaging data repository. Access requests will be granted depending on appropriate regulatory and institutional approvals upon contacting the corresponding author.
