# Supplementary material for: Biomarkers for site-specific response to neoadjuvant chemotherapy in epithelial ovarian cancer: relating MRI changes to tumour cell load and necrosis
Source: Br J Cancer. 2021 Jan 4;124(6):1130–7. doi: 10.1038/s41416-020-01217-5 (PMC7961011; doi:10.1038/s41416-020-01217-5)
Supplement: Supplementary file 1 — Supplementary Material [file 41416_2020_1217_MOESM1_ESM.docx]

**Supplementary Figure 1**

**Supplementary Figure 1:** Flowchart showing participant and lesion numbers available for analysis. Participants were enrolled at four hospitals, with imaging data available from all four hospitals, and imaging and correlated pathology data available from three of four hospitals. For the 40 participants with MRI examinations at baseline and after cycle three or four: the median interval between baseline MRI and starting chemotherapy was 5 days (range 0 to 14 days) (date of first baseline taken in patients with two baseline examinations). Platinum-based chemotherapy was administered on day one of a 21-day cycle in all participants, with a median interval between day one of the third or fourth cycle and the post-treatment MRI was 20 days (range 15 to 39 days).

**Supplementary Table 1:** MRI protocols from four centres.

|  | Site 1 | Site 2 | Site 3 | Site 4 |
| --- | --- | --- | --- | --- |
| Manufacturer | Siemens Healthcare, Erlangen, Germany | GE Healthcare, Waukesha, WI, USA | GE Healthcare, Waukesha, WI, USA | Siemens Healthcare, Erlangen, Germany |
| Model | MAGNETOM Avanto | Discovery MR450 | Optima MR450w | MAGNETOM Avanto Fit |
| Field strength/T | 1.5 | 1.5 | 1.5 | 1.5 |
| Maximum gradient amplitude/mT m^−1^ | 45 | 50 | 34 | 45 |
| Maximum slew rate/T m^−1^ s^−1^ | 200 | 200 | 150 | 200 |
| Receive coil (s) | 2 × anterior body matrix and posterior spine matrix | body array | body array | 2 × anterior body matrix and posterior spine matrix |
| Participant position | Feet first supine | Feet first supine | Feet first supine | Head first supine |
| Slice orientation | Axial | Axial | Axial | Axial |
| Slice thickness/mm | 6 | 6 | 6 | 6 |
| Slices per station | 26 | 26 | 26 | 26 |
| Number of stations | 3 | 3 | 3 | 3 |
| PE direction | AP | AP | AP | AP |
| Diffusion-weighted MRI | | | | |
| Sequence | Single-shot EPI | Single-shot EPI | Single-shot EPI | Single-shot EPI |
| FOV (read)/mm | 380 | 380 | 380 | 380 |
| FOV (phase)/mm | 332 | 334 | 334 | 332 |
| Acquired matrix (read) | 128 | 128 | 128 | 128 |
| Reconstructed matrix (read) | 256 | 256 | 256 | 256 |
| Acquired pixel size/mm x mm | 3.0 × 3.0 | 3.0 × 3.0 | 3.0 × 3.0 | 3.0 × 3.0 |
| Reconstructed pixel size/mm x mm | 1.5 × 1.5 | 1.5 × 1.5 | 1.5 × 1.5 | 1.5 × 1.5 |
| Echo time (TE)/ms | 75 | 81 | 75 | 76 |
| Repetition time (TR)/ms | 8000 | 8000 | 8000 | 8000 |
| Receive bandwidth | 1776 Hz/pixel | Receiver bandwidth ± 125 kHz (pixel bandwidth 1953 Hz/pixel) | Receiver bandwidth ± 125 kHz (pixel bandwidth 1953 Hz/pixel) | 1562 Hz/pixel |
| Number of signal averages (NSA) | 4 | 4 | 4 | 4 |
| Parallel imaging | GRAPPA, reduction factor 2; 36 ACS lines | ASSET, reduction factor 2 | ASSET, reduction factor 2 | GRAPPA, reduction factor 2; 36 ACS lines |
| Partial Fourier | no | yes | yes | no |
| Fat suppression | SPAIR | Water-selective excitation | Water-selective excitation | SPAIR |
| Diffusion gradient scheme | bipolar | DSE | monopolar | bipolar |
| Number of diffusion directions | 3 | 3 | 3 | 3 |
| Diffusion encoding scheme | 3-scan trace | ALL | ALL | 3-scan trace |
| Diffusion-weighted MRI series used for analysis | Trace | Trace | Trace | Trace |
| Acquired b-values/s mm^−2^ | 0, 100, 500, 900 | 0, 100, 500, 900 | 0, 100, 500, 900 | 0, 100, 500, 900 |
| Breathing instructions | Free breathing | Free breathing | Free breathing | Free breathing |
| Navigator/gating | none | none | none | none |
| Acquisition time per station | 5 mins 44 s | 5 mins 28 s | 5 mins 28 s | 5 mins 46 s |
| T_1_-weighted MRI | | | | |
| Sequence | 2D gradient echo (FLASH) | 2D gradient echo (FSPGR) | 2D gradient echo (FSPGR) | 2D gradient echo (FLASH) |
| FOV (read)/mm | 380 | 380 | 380 | 380 |
| FOV (phase)/mm | 332 | 334 | 334 | 332 |
| Acquired matrix (read) | 256 | 256 | 256 | 256 |
| Acquired pixel size/mm x mm | 1.5 × 1.5 | 1.5 × 1.5 | 1.5 × 1.5 | 1.5 × 1.5 |
| Echo time (TE)/ms | 4.82 | 4.2 | 4.2 | 4.82 |
| Repetition time (TR)/ms | 139 | 139 | 139 | 139 |
| Fat suppression | None | None | None | None |
| Flip angle/° | 70 | 70 | 70 | 70 |
| Breathing instructions | 2 breath-holds | 2 breath-holds | 2 breath-holds | 2 breath-holds |
| Acquisition time per station | 35 s (17.5 s per breath-hold) | 34 s (17 s per breath-hold) | 44 s (22 s per breath-hold) | 35 s (17.5 s per breath-hold) |
| T_2_-weighted MRI | | | | |
| Sequence | Single-shot turbo spin echo (HASTE) | Single-shot turbo spin echo (SSFSE) | Single-shot turbo spin echo (SSFSE) | Single-shot turbo spin echo (HASTE) |
| FOV (read)/mm | 380 | 380 | 380 | 380 |
| FOV (phase)/mm | 332 | 380 | 334 | 332 |
| Acquired matrix (read) | 256 | 256 | 256 | 256 |
| Acquired pixel size/mm x mm | 1.5 × 1.5 | 1.5 × 1.5 | 1.5 × 1.5 | 1.5 × 1.5 |
| Echo time (TE)/ms | 90 | 90 | 92 | 92 |
| Repetition time (TR)/ms | 1500 | 1500 | 1500 | 1500 |
| Fat suppression | None | None | None | None |
| Breathing instructions | 2 breath-holds | 2 breath-holds | 2 breath-holds | 2 breath-holds |
| Acquisition time per station | 39 s (19.5 s per breath-hold) | 38 s (19 s per breath-hold) | 38 s (19 s per breath-hold) | 39 s (19.5 s per breath-hold) |

EPI = echo planar imaging, FOV = field of view, PE = phase encoding, AP = anterior-posterior, TE = echo time, TR = repetition time, NSA = number of signal averages, GRAPPA = generalized autocalibrating partially parallel acquisition, ASSET = array spatial sensitivity encoding technique, SPAIR = spectral adiabatic inversion recovery, DSE = double spin-echo, FLASH = fast low-angle shot, FSPGR = fast spoiled gradient echo, HASTE = half-Fourier single-shot turbo spin echo, SSFSE = single-shot fast spin echo.

**Supplementary Table 2:** Study participant demographics, clinical characteristics, and chemotherapy schedules.

| Variable |  |
| --- | --- |
| Number of participants | 47 |
| Sex  n women (%) | 47 women (100%) |
| Age / years  median (IQR) | 61 (57-70) |
| Histological subtype  n (%) | serous 47 (100%) |
| Current chemotherapy regimen | |
| Carboplatin monotherapy  n (%) | 3 (6%)  *weekly 0 (0%)*  *3-weekly 3 (100%)* |
| Carboplatin and paclitaxel  n (%) | 44 (94%)  *weekly carboplatin 1 (2%)*‡  *3-weekly carboplatin 43 (98%)*  *weekly paclitaxel 9 (20%)*  *3-weekly paclitaxel 35 (80%)* |
| Also receiving bevacizumab #  n (%) | 5 (11%) |

‡ participant in ICON8 study;

# bevacizumab given during neoadjuvant chemotherapy.

IQR = interquartile range.

**Supplementary Table 3:** Site-specific repeatability of solid tumour volume and ADC_median_ in epithelial ovarian cancer.

| Tumour site | n | 95 % LoA for Volume [95 % CI]  / cm^3^ | 95% LoA for ADC_median_ [95 % CI]  / 10^-5^ mm^2^ s^-1^ |
| --- | --- | --- | --- |
| Ovary | 20 | -19.2 [-26.8, -11.5] to 17.9 [10.3, 25.5] | -10 [-14, -6] to 9 [5, 13] |
| Peritoneum | 52 | -5.7 [-7.1, -4.4] to 5.4 [4.0, 6.8] | -13 [-17, -10] to 16 [12, 19] |
| Omentum | 23 | -43.2 [-59.5, -26.9] to 42.3 [26.0, 58.6] | -17 [-24, -11] to 17 [11, 24] |
| Lymph node | 28 | -3.2 [-4.1, -2.3] to 2.2 [1.3, 3.2] | -27 [-35, -19] to 21 [13, 29] |

n = number of lesions,

ADC = apparent diffusion coefficient (where ADC_median_ is defined as the median ADC of all fitted voxels in a lesion),

LoA = limits of agreement,

CI = confidence interval.
